# Evaluation of Neurogenic Potential of Human Umbilical Cord Mesenchymal Cells; a Time- and Concentration-Dependent Manner

**DOI:** 10.6091/ibj.1452.2015

**Published:** 2015-04

**Authors:** Seyed Hassan Eftekhar-Vaghefi, Leila Zahmatkesh, Parvin Salehinejad, Shahin Totonchi, Ali Shams-Ara

**Affiliations:** 1 *Neuroscience Research Center, Kerman University of Medical Sciences, Kerman, Iran; *; 2 *Dept. of Anatomy,Afzalipour School of Medicine, Kerman University of Medical Sciences, Kerman , Iran;*; 3 *School of Medicine, Yazd University of Medical Sciences, Yazd, Iran*

**Keywords:** Cell differentiation, Neural stem cells, Retinoic acid

## Abstract

**Background:**

Retinoic acid as one of the most important regulators for cell differentiation was examined in this study for differentiation of human umbilical mesenchymal cells (hUCM).

**Methods:**

After isolation, hUCM were evaluated for mesenchymal stem cell properties by flow cytometry and alkaline phosphatase assay. Also, doubling time of the cells and their differentiation potential into adipogenic and osteogenic cells were tested. hUCM were then cultured with different concentrations of retinoic acid, and on days 1, 7, and 12, the percentage of differentiated cells was determined by immunostaining for nestin, anti-microtubule associated protein 2 (MAP_2_), glutamic acid decarboxylase (GAD), and gamma-aminobutyric acid (GABA) markers.

**Results:**

The isolated cells were negative for the hematopoietic markers and positive for the mesenchymal markers. They showed the population doubling time 60 *± *3 hours and differentiated into osteogenic and adipogenic cells. A descending trend in nestin and an ascending trend in MAP_2_, GAD, and GABA expression were observed from the first day until the last day between different concentrations of retinoic acid.

**Conclusion:**

hUCM cells may have the potential to differentiate into neural cells in the presence of different incubation period and concentration of retinoic acid.

## INTRODUCTION

Mesenchymal stem cells (MSC) are commonly isolated from bone marrow [1]. However, harvesting bone marrow is an invasive and a painful procedure, and the proliferation/differentiation capacity declines with age [2]. Other than this source, MSC have been identified in a variety of tissues, such as adipose tissue, peripheral blood, spleen, brain, synovial fluid, dermis, muscle, dental pulp, skin and umbilical cord that differentiate along several mesenchymal lineages [3]. Of course, there are significant differences in the proliferation and differentiation abilities among these MSC. The umbilical cord, which is an organ discarded after birth, does not require an invasive procedure with ethical concerns to collection of the cells [3]. Human umbilical cord mesenchymal (hUCM) cells possess stem cell properties, such as self-renew, proliferation, and multipotential differentiation. These cells may be potential sources as cell therapy for neurodegenerative diseases [4].

Retinoic acid is well known as the biological activator of vitamin A and plays an important role during embryogenesis [5]. Embryonic stem cells can be induced to differentiate into neurons and glia cells by applying a modified medium enriched with retinoic acid [6]. It has been shown that related protocols with retinoic acid treatment increased the yield of neural derivatives, including glutamatergic neurons and motor neurons [7-9]. Gama-aminobutyric acid (GABA) is the primary inhibitory neurotransmitter in the central nervous system. A decrease in GABAergic neuro-transmission is associated with many severe neurological disorders, including epilepsy, schizo-phrenia, Huntington’s disease, chronic pain, anxiety, and other mood disorders [10-12]. Fu *et al.* [13] have previously demonstrated that hUCM could be induced to differentiate into neuron-like cells. Salehinejad *et al.* [14] used different cocktails consisting of retinoic acid for neural differentiation of hUCM, while retinoic acid was not used in a time- and concentration-dependent manner to differentiate these cells. 

In the present study, the isolated hUCM exposed to various concentrations of retinoic acid transformed into GABAergic neurons *in vitro*. The goal of this study was to determine the percentage of differentiation of hUCM into neuron-like cells in defined times when different concentrations of retinoic acid were applied to these cells.

## MATERIALS AND METHODS

***Isolation of the human umbilical*** ***cord*** ***mesenchymal cells.*** Fresh human umbilical cord was obtained during cesarean section after receiving an informed consent from mothers who referred to Gynecology Ward in the Afzalipour Hospital in Kerman University of Medical Sciences (Kerman, Iran). Umbilical cord was collected in Hanks’ balanced salt solution and transferred to laboratory for tissue processing. After removal of blood vessels, the Wharton's jelly tissue was removed and divided into 2-3 mm^3^ pieces. The pieces were explanted onto 35 × 10 mm Petri dishes and cultured in DMEM/F12 supplemented with 10% fetal bovine serum, 100 IU/ml penicillin, 50 μg/ml streptomycin sulfate and 2.5 μg/ml amphotericin B in 5% CO_2_ at 37ºC for 2 weeks. After cell buds observation, Wharton's jelly pieces were removed, and cell culture was continued to 80% confluency.

***Flow cytometry*** ***.*** For detection of mesenchymal characteristics, after the fourth passage, the cells were prepared at a concentration of 10^5^ cells/ml in DMEM/F12 with 10% FBS. The cells were incubated at 4^ο^C for 15 minutes with a 1:9 dilution of normal goat serum in PBS to block non-specific binding of the antibody. The cells were then labeled with the following antibodies: FITC-conjugated anti-CD34, FITC-conjugated anti-CD44 (Chemicon, USA), FITC-conjugated anti-CD45 (Ediscience, USA), PE-conjugated anti-CD90 (Dako, Denmark) and PE-conjugated anti-CD105 (R and D, USA) for 1 hour. The cells were analyzed using FACSCalibur (Becton Dickenson, Pharmingen, San Diego, CA, USA) machine. At least 10,000 events were recorded for each sample and data were analyzed using WinMDI 2.9 software (USA). 

***Alkaline phosphatase assay*** *.* For detection of osteogenic ability, the hUCM from the fourth passage were grown on sterile glass slides for 2 weeks. The medium was refreshed daily, and alkaline phosphatase activity was detected using an ALP Kit (R-87 Sigma, Germany) according to the manufacturer’s instructions. After exposure to the substrate, a dark red product confirmed alkaline phosphatase activity. The cells were then counterstained with hematoxylin and mounted and photographed using an Olympus DP71 digital camera (Japan) attached to an IX71 inverted microscope (Japan).

***Osteogenic and adipogenic differentiation*** *.* For detection of mesenchymal properties, the fourth passage of the hUCM was grown on glass coverslips (confluency of 70%). Osteogenic medium, which was composed of DMEM/F12 containing 10% FBS, 10 nM dexamethasone, 50 μg ascorbic acid, and 10 mM β-glycerophosphate, was added to the cells. Medium was replaced every three days for a three-week period. After 21 days, the cells were fixed with methanol at room temperature for 10 minutes and then stained with Alizarin red for 20 minutes. Finally, they were washed with distilled water and drained. For adipogenic differentiation, the hUCM were treated with 100 nM dexamethasone, 50 μg ascorbic acid in DMEM/F12 containing 10% FBS. Medium was replaced every three days for a three-week period. On day 21, cells were fixed in 10% buffered formalin at room temperature for 30-60 minutes and stained with 10% Oil red O for 30 minutes. Then, they were washed with water, and hematoxylin was used as nuclear counterstain.

***Doubling time assay.*** For detection of cell proliferation ability, mean doubling time of the hUCM was calculated using the obtained cell counts from day one through day seven, and the procedure was repeated with cells from three separate cords. aliquots of 4 ×10^4^ hUCM cells were plated into 35-mm Petri dishes [15] to obtain the doubling time,. On days 1 through 7 of culture, the dishes were trypsinized, and the cells were counted. The total number of live cells was obtained at each time point by staining with 0.4% Trypan blue and an improved Neubauer hemocytometer (Germany). Doubling times were calculated using the following equation: CD = l n (Nf/Ni)/ln2 [16], and DT = CT/CD, that Nf was final number of cells, Ni was initial number of cells, and DT, CT, and CD were the cell doubling time, cell culture time and cell doubling number, respectively.

***Neural differentiation.*** At the fourth passage, the hUCM cells were seeded at a density of 1 × 10^4^ cells/ml onto sterilized glass slides. After one-hour incubation at 37^○^C, the cells were treated with 10^-4^ [17] and 10^-6 ^M [18] dosages of all trans-retinoic acid (Sigma, R 2625) for three days. Then retinoic acid was removed from the media, and the cells remained until the other nine days, while the media were refreshed every two days. 

***Immunocytochemistry.*** For detection of neural differentiation rates, hUCM were fixed with 4% paraformaldehyde in 0.1 M phosphate buffer for 20 minutes and washed with 0.1 M phosphate buffer. They were then treated with a blocking solution (10% H_2_O_2_) for 20 minutes to prevent non-specific antibody-antigen binding. The cells were then incubated with primary antibodies: mouse anti-nestin antibody (1:200, Chemicon, USA), mouse anti-microtubule associated protein 2 (MAP_2_) antibody (1:1,000, Sigma, Germany), mouse anti-GAD (glutamic acid decarboxylase) antibody (1:2,000, Chemicon, USA), and anti-rabbit GABA antibody (1:100, Sigma, Germany) at room temperature for 45 minutes. After washing in Tris-buffered saline, the cells were incubated with secondary antibodies at room temperature for 1 hour. Then the slides were counterstained with hematoxylin (Dako, Denmark), and negative controls were considered by omitting the primary antibody. Two hundred cells in random fields were examined in each culture slide at ×10 magnification. All slides were studied using an optical microscope (Nikon-YS100, Japan). The fraction of positive cells was calculated by counting 10 non-overlapping microscopic fields for each slide in at least three separate experiments.

***Statistical analysis.*** The statistical analyses were carried out using two-way ANOVA with Tukey’s multiple comparisons. For each parameter, the significant level was considered *P* ≤ 0.05.

## RESULTS

**Fig. 1:**
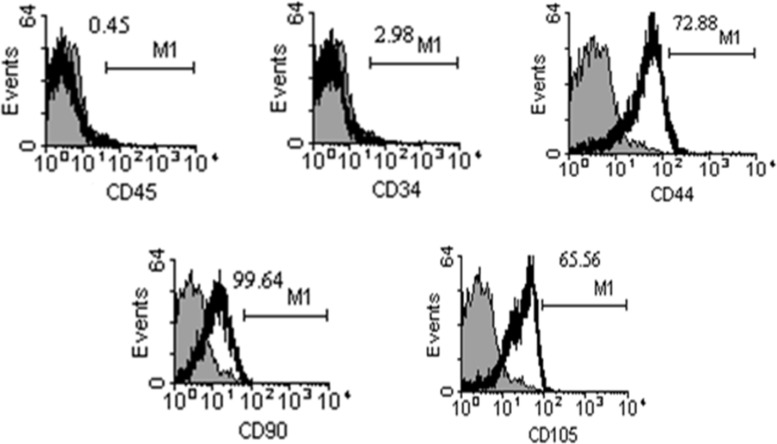
Flow cytometry results. The gray and black histograms show the isotype control-stained cells as well as the antibody-stained cells, respectively. Human umbilical mesenchymal cells were negative for hematopoietic markers (CD34 and CD45) and positive for mesenchymal stem cell markers (CD44, CD90, and CD105).

***Flow cytometry.*** The isolated cells were negative for the hematopoietic cell-surface markers CD34 and CD45, while the MSC markers CD44, CD90, and CD105 were expressed positively in the cells (Fig. 1).

**Fig. 2:**
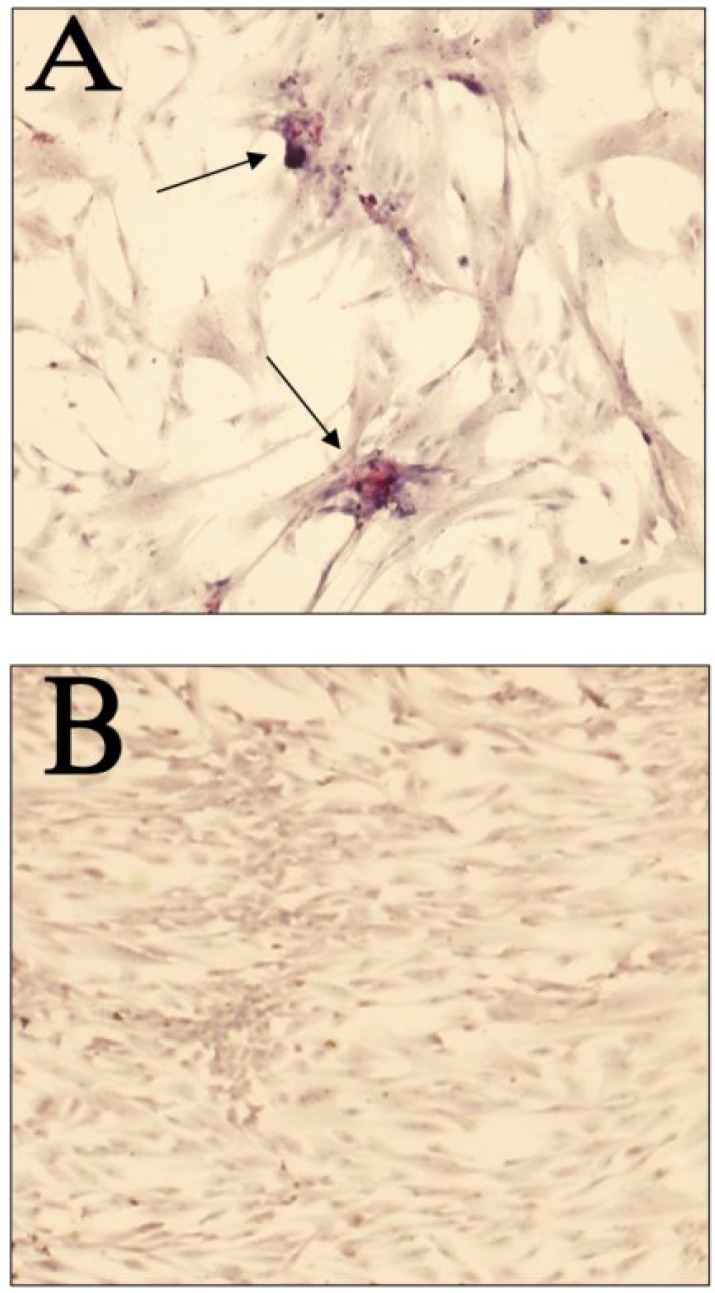
Alkaline phosphatase activity in hUCM. (A) Arrows show the cell colonies were positively stained with ALP (dark red color). (B) No ALP-positive cells were detectable in hUCM treated with levamisole. Scale bars are 25 and 100 μm for (A) and (B), respectively.

***Alkaline phosphatase*** ***.*** hUCM cells showed positive stained pattern for ALP. The hUCM cells were maintained in culture until colony formation was observed. The reaction product was most intense within the colonies (Fig. 2).

**Fig. 3:**
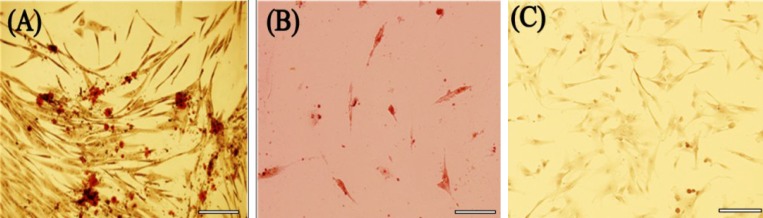
Osteogenic and adipogenic differentiation of hUCM. (A) Calcium deposition and osteoid formation as shown by Alizarin red. (B) Their adipocytic phenotypes were signaled by the appearance of tiny intracytoplasmic lipid droplets with Oil red O. (C) Negative control showed no evidence of staining. Scale bars, 50 μm.

***Osteogenic and adipogenic differentiation.*** The results of osteogenic differentiation showed that almost 30-40% hUCM differentiated into these cells. The staining was reddening of the organic extracellular materials including calcium (Fig. 3A). hUCM differentiation showed that about 10-20% these cells differentiated to adipogenic cells. Lipid droplet appeared at the third and fourth days and got oversized little by little at the end of the differentiation period (Fig. 3B).

**Fig. 4:**
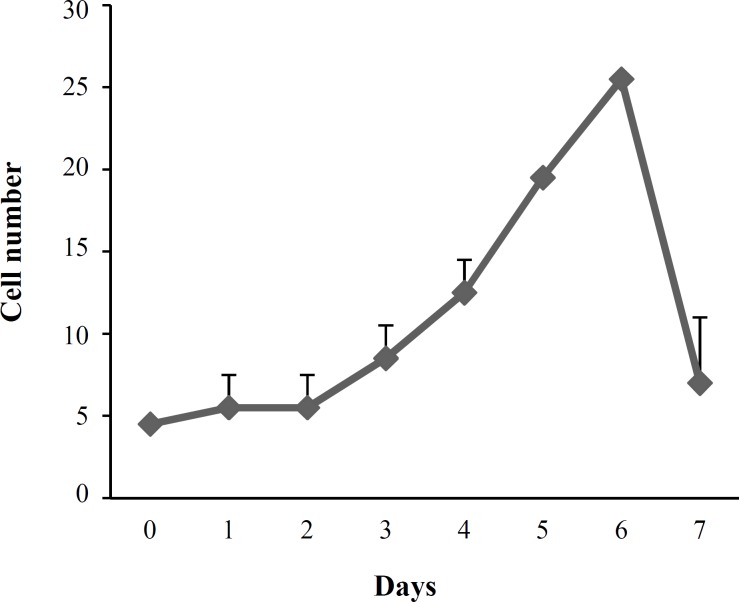
Doubling time in hUCM. Accumulated data obtained from cultures showed that the lag phase lasts around the first day, leading to five days of log phase. The sharp cell number was about 26 × 10^4 ^in the six^th^ day and after it, the cell number started to decrease.

***Doubling time. ***The increase in cell concentration (y axis) through culture days (x axis) took almost six days in the cells when cells grew exponentially (Fig. 4). The mean population doubling time of the hUCM was 60 *± *3 hours (mean *± *SE).

**Fig. 5:**
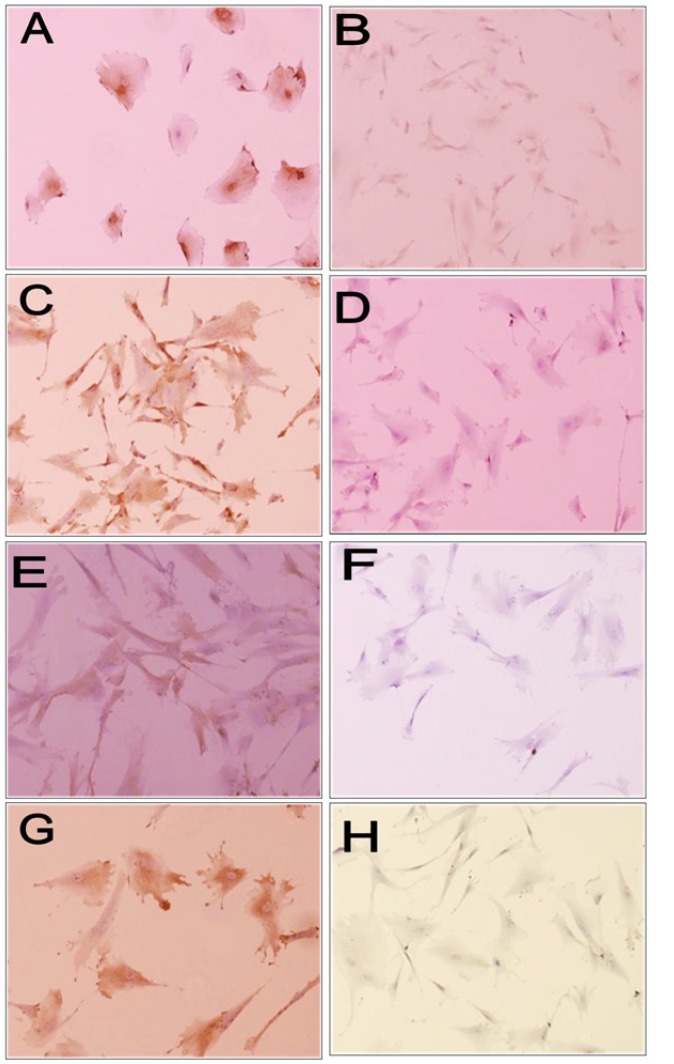
Differentiation of hUCM to neuronal lineage. (A) Nestin-positive cells that located around nuclei of differentiated hUCM; (B) Nestin-negative control; (C) MAP_2_-positive cells that appeared as several patch within perikarya in induced cells at later stage; (D) MAP_2_ negative control; (E) GAD-positive cells; (F) GAD negative cells; (G) GABA-positive cells; (H) GABA negative cells. Scale bars, 25 μm for B, C, D, E, F and H; 50 μm for A and G.

**Fig. 6:**
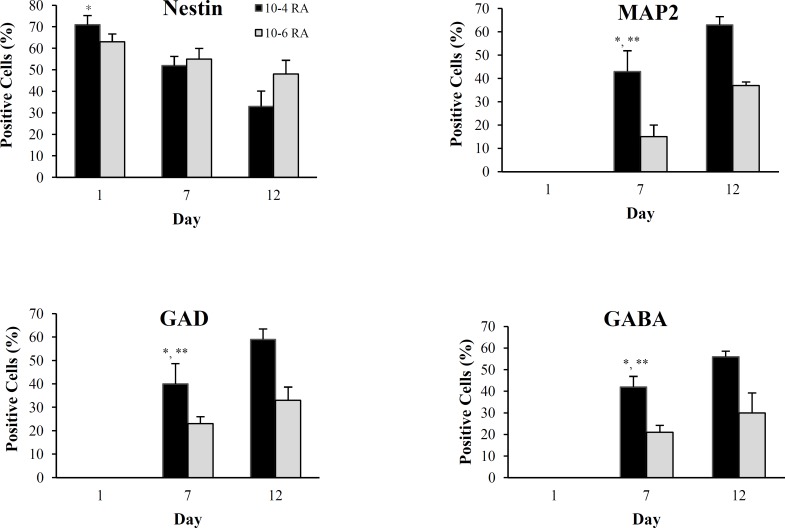
Expression of specific neural markers in various times in two concentrations. *P < 0.05, differences among 1, 7, and 12 days in a marker. **P < 0.05, differences between two concentrations in MAP_2_, GAD, and GABA markers

***Neural differentiation.*** Under defined conditions, induced hUCM expressed special neural markers in a time- and concentration-dependent manner (Fig. 5). At 10^-6^M concentration of retinoic acid, 63 ± 3.7% of cells were positive for nestin on day one, while about 55 ± 5% of the cells on day seven and approximately 48 ± 3.7% of the cells on day 12 were positive. Reduction in the number of positive cells from day 1 through day 12 was not statistically significant (*P *> 0.05). The nestin expression reduction was more prominent in higher concentration of retinoic acid (10^-4^) so that at the first day 71 ± 4.3% of cells was labeled by anti-nestin, that this amount reached 52 ± 4.3% and 33 ± 7.2% by the days 7 and 12, respectively. This reduction process in the above mentioned days was statistically significant (*P* < 0.05). HUCMs clearly showed no evidence of MAP_2_ expression in the undifferentiated state and on day one. However, on day seven, this level reached 43 ± 8.96% at concentration of 10^-4^M, and 15± 5% at a concentration of 10^-6^ M. On day 12, 63± 3.5% of cells were positive at a concentration of 10^-4^ and 37% at a concentration of 10^-6 ^(*P* < 0.05). These data expressed that MAP_2_-positive cells were increased significantly in the course of time, and this process is more accelerated by 10^-4^ retinoic acid. To assess neuro-transmitter phenotypes, we used immunecytochemistry staining with antibodies of GABA and GABA synthetic enzyme GAD, as a marker for GABAergic neurons [19]. On day seven, nearly a mass of 42 ± 4.9% of cells at a concentration of 10^-4^ M and 21 ± 3.2% of cells at a concentration of 10^-6^ M were positive for GABA, while 40 ± 8.7% of the cells at concentration of 10^-4^ M and 23 ± 3% at the concentration of 10^-6^ M were GAD positive. These cells in the concentration of 10^-4^ M reached a level of 56 ± 2.6 for GABA and 59 ± 4.5 for GAD expression on day 12. Similarly, the cells at concentration of 10^-6^M reached a level of 30 ± 9.2% for GABA and 33 ± 5.7% for GAD expression on day 12. Considering *P* < 0.05, the presence of GABA and GAD antibody in both concentrations was meaningful. This process speeds up at high concentration (10^-4^ M) (Table 1). Figure 6 shows the percentage of antibodies expressed on different days at 10^-4^ and 10^-6 ^M concentrations of retinoic acid, which were figured out by ANOVA (*P* < 0.05).

## DISCUSSION

Knowledge about how initially stem cell populations can give rise to diverse neural cell types *in vitro*, and how we can direct stem cells to produce definite cell types may help us to better understand the *in vivo *developmental processes and design potential therapeutic approaches to neurological disorders [18]. Thus, this *in vitro* study was planned to determine the effect of two concentrations of retinoic acid on hUCM differentiation into neural cells in various times. We found that 10^-4^ M concentration of retinoic acid induced hUCM cells to express the highest percentage of the neural markers. 

We used the human umbilical cord as a new source of stem cells. The human umbilical cord contains a mesenchymal precursor cell population that gives rise to the Wharton's jelly connective tissue. These cells would most likely be located closest to the vascular region [20]. hUCM can be easily obtained and processed as compared with embryonic and bone marrow stem cells, and its compatibility with the immune system of the body is confirmed and can live in the host body for a long time [13]. 

Retinoic acid influences on neural differentiation by mechanisms which still have remained undefined. Retinoic acid can modulate the differentiation properties of cells into various cell types, including skeletal muscle cells, adipocytes, cardiomyocytes, vascular smooth muscle cells, and neural cells [21]. Cell differentiation *in vitro* and *in vivo* relates to morphologic changes based on regulation of intermediate filament protein [22]. Therefore, region- specific transcription factors get activated in stem cell populations induced by retinoic acid. The homo-domain transcription factors (*emx2, dlx2*, and* hoxb2) *and bHLH* (ngn2, *and *mash1)*, which are specific for different central nervous system domains, can be expressed in the induced cells. The cells produced diverse neuronal phenotypes during the *in vitro *differentiation. In neuron-rich cell cultures, glutamatergic, GABAergic, serotonergic, and cholinergic markers (VGlut1, VGlut2, Gad65, Gad67, VGAT, GABA, 5-HT, and chat) at gene expression and/or protein level can be detected [23]. Similarly to the *in vivo *developmental processes, astroglial cells emerged only following the main neuron forming period in course of the *in vitro *differentiation of the cells [24].

**Table 1:** Expression of specific neural markers in various times in two concentrations

**Antibodies**		**10** ^-4^ ** RA**				**10** ^-6 ^ **RA**		
		**Day 1 (%)**	**Day 7 (%)**	**Day 12 (%)**		**Day 1 (%)**	**Day 7 (%)**	**Day 12 (%)**
Nestin		71 ± 4.3	52 ± 4.3	33 ± 7.2		63 ± 3.7	55 ± 5.0	48 ± 6.5
MAP2		0	43 ± 8.9	63 ± 3.5		0	15 ± 5.0	37 ± 1.5
GAD		0	40 ± 8.7	59 ± 4.5		0	23 ± 3.0	33 ± 5.7
GABA		0	42 ± 4.9	56 ± 2.6		0	21 ± 3.2	30 ± 9.2

Data are expressed as mean ± SEM. RA, retinoic acid

Retinoic acid can differentiate the cells into different cell types in an incubation time- and concentration-dependent manner [21]. It has been shown that the addition of 0.5 to 1 μM retinoic acid suppresses non-neural differentiation and instead results in a high proportion of cells becoming neurons or astrocytes [25]. Hence, we used 10^-4^ and 10^-6 ^M concentrations of retinoic acid to differentiate hUCM into neurotic cells. Also, we used particular neurotic antibodies, including nestin antibody to detect neuronal progenitor cells [24], MAP_2_ antibody to detect mature neurons, GAD antibody to detect GABAergic immature neurons [17], and GABA antibody to detect GABAergic mature neurons [19].

Nestin is an intermediate filament protein and a marker for neuronal progenitor cells that is expressed in dividing cells during the early stages of development in the central and peripheral nervous systems. Nestin is preferentially located around nuclei of differentiated hUCM [24, 26]. Karahuseyinoglu *et al.* [26] showed that nestin expression was exclusively confined to neuronally induced cells. Therefore, in order to determine the neuroepithelial cells and neuronal progenitor cells, we performed immunostaining with anti-nestin [24]. During three days of retinoic acid exposure and one day after removing retinoic acid, we observed the expression of nestin 71% and 63% (in 10^-4^ and 10^-6 ^M concentrations, respectively) in the hUCM. In the control group, nestin-positive cells were not observed. Gharibani *et al*. [27] used 10^-5^ M retinoic acid for three days. After removal of the retinoic acid, 72% of the cells were nestin positive at day one. This amount was decreased to 25% until day 11 [17]. In this study, the expression of nestin levels with 10^-4^ M retinoic acid on day 12 reached to 33% and with 10^-6^ M retinoic acid reached to 48%. Also, nestin after differentiation became down-regulated [28]. From these data, it can be inferred that the number of the cells switched from stem cells to differentiated cells has increased significantly during this period (day 1-day 12), and the nestin expression has a close relationship to the time.

MAP_2_, a dendrite-specific protein prominent in mature neurons, was positive in cell to cell contact [26]. Chatizi *et al.* [17] added retinoic acid for three days and also bFGF, B27, and Neurobasal in culture media for three days. They resulted that one day after withdrawal of retinoic acid, at none of the concentrations (10^-4^ and 10^-5 ^M), the cells did not express MAP2. However, in our study, at day seven after withdrawal of retinoic acid, the rate of expression of this marker reached to 43% (10^-4^ M) and 15% (10^-6 ^M) (*P *< 0.05). On day 12, 63% (10^-4^ M) and 37% (10^-6 ^M) of these cells were MAP2 positive (*P* < 0.05). It is probable that treatment with higher concentrations of retinoic acid promoted acquisition of polarity for the formation of neural outgrowths. By comparing the results of this study with those of Fraichard *et al*. [29], who observed gradual increase in the expression of MAP2 from 25% to 90%, we can conclude that if retinoic acid is used alone as differentiating agent, cells can gradually differentiated, and with passage of time, the percentage of differentiated cells increase, whereas when using retinoic acid in combination with other growth factors, the rate of differentiation increases and a higher number of cells differentiate in a shorter time period. The proportion of MAP2-positive cells in this study was 63%, while it was 90% in other studies. The heterogeneity of the hUCM cells is one of the reasons for difference of MAP2 expression among the studies.

GAD, the synthetic enzyme of the neurotransmitter y-aminobutyric acid (GABA), has been localized within the axon terminals of specific neurons, in the somata and dendrites of neurons [30]. In this investigation, on day seven, a large population of cells was GAD positive, approximately 40% in 10^-4 ^M and 23% in 10^-6^M concentrations, and it reached on day12 to 59% (10^-4^M) and 33% (10^-6^M) that was significant at both concentrations. Therefore, when using higher concentrations of retinoic acid, increase cell differentiation would be higher. Moreover, expression of GAD-positive cells was progressive which was consistent with Chatzi *et al.* [17] findings. This data suggests that both concentrations can cause differentiation of hUCM, but at 10^-4 ^M retinoic acid, differentiation occurs faster and can produce more neuronal cell, especially pre-GABAergic cells.

Treatment with retinoic acid promotes neuronal maturation and produces neurons with specific phenotypic properties, such as GABAergic neurons [31]. In mammals, GABA is synthesized from glutamate by two glutamate decarboxylases and transported by vesicular GABA transporter. However, this GABA cannot cross the blood-brain barrier effectively. Certainly, in the central nervous system, crossing GABA in the blood depends on its production from local neurons. In this direction, investigators have tried to product GABAergic cell [32]. In this study, we have been successful at inducing the differentiation of GABAergic neurons and showed that retinoic acid in a time- and concentration-dependent manner causes the induction of MSC of the Wharton's jelly and their differentiation into GABAergic neurons. The number of GABA-positive cells was 42% (10^-4^ M) and 21% (10^-6^M) on day 7, and this rate reached 56% and 30%, respectively on day 12 (*P* < 0.05). Chatizi *et al*. [17] showed 25% of GABA-positive neurons on day 4 and 90% on day 7. However, in our study, only 56% of the cells differentiated into GABA-positive neurons on day 12, which could be because the source of cells was different from Chatizi *et al.* [17]. Of course, FBS can affect the study results because it contains a significant level of retinoic acid. In an interesting study performed by Kim *et al.* [31] to differentiate neuronal embryonic stem cells, serum-free medium (containing 0.5 mM retinoic acid) was added for 4 days and ultimately, 22% of the cells became GABA positive. Therefore, the percentage of cells expressing GABA depended not only on retinoic acid concentrations but also on the rate of serum use as well as on the days that the cells were placed in the presence of retinoic acid.

We conclude that differentiation of hUCM into neuron-like cells in defined times is highly dependent on the concentration of retinoic acid. When 10^-4 ^M concentration was applied, more than half of the cells differentiated, but less percentage of the cells differentiated at 10^-6^ M concentration. The expression of MAP_2_, GAD, and GABA increased in time progress. However, we had some limitations for preparation of other neural markers in this study. It was better if we examined astrocyte and oligodendrocyte markers by using retinoic acid in various concentrations and times.
